# Psychological mechanisms underlying medical students' continued use of DeepSeek: an explanatory sequential mixed-methods study

**DOI:** 10.3389/fpsyg.2026.1875736

**Published:** 2026-07-08

**Authors:** Shikai Chen, Xinling Wu, Ying Ma, Yuanyuan Zhang, Hao Zhang, Xiaofei Bian

**Affiliations:** 1 School of Public Health, Dalian Medical University, Dalian, Liaoning, China; 2 The Department of Mental Health and Psychology, Dalian Medical University, Dalian, Liaoning, China; 3 School of Marxism, Dalian Medical University, Dalian, Liaoning, China; 4 Department of Pediatrics, Dalian Medical University, Dalian, Liaoning, China

**Keywords:** continuance intention, DeepSeek, Generative AI, medical education, mixed-methods study, task-technology fit

## Abstract

**Background:**

Generative AI is becoming widely used in medical education, but what drives medical students to continue using domestic tools such as DeepSeek after initial adoption remains poorly understood.

**Purpose:**

This study examined the psychological mechanisms underlying medical students continued use of DeepSeek, with particular attention to how cognitive appraisals, satisfaction, and task fit shaped continuance in real learning contexts.

**Methods:**

An explanatory sequential mixed-methods design was used. In the quantitative phase, 630 valid questionnaires were analyzed using structural equation modeling to test a continuance pathway centered on cognitive appraisal, satisfaction, and behavioral intention, while interview data were used to explain unexpected and nonsignificant quantitative findings.

**Results:**

System quality and subjective norm positively affected perceived ease of use, while subjective norm and expectation confirmation positively affected perceived usefulness. Perceived ease of use and perceived usefulness both increased satisfaction, and satisfaction was the strongest predictor of continuance intention. Task-technology fit also positively influenced continuance intention, which strongly predicted actual continued use. Technology characteristics and task characteristics both improved task-technology fit. By contrast, information quality negatively affected perceived ease of use, subjective norm negatively affected satisfaction, and privacy concerns and expectation confirmation did not significantly affect continuance intention or satisfaction. Students mainly continued using DeepSeek because it was easy to access, helpful for academic writing and exam preparation, and suited to some specialized tasks; their primary concerns were unstable performance, inaccurate outputs, future pricing, and data security.

**Conclusion:**

Continued DeepSeek use followed a cognitive-affective-behavioral sequence: perceived ease of use and usefulness drove satisfaction, which in turn predicted continuance intention (β = 0.769), while task-technology fit provided an independent behavioral pathway (β = 0.157), together accounting for actual continued use (β = 0.732).

## 1 Introduction

Since DeepSeek entered public use in early 2025, it has quickly attracted attention among Chinese university students. In medical education, students have begun to use it for literature retrieval, case-based reasoning, and examination preparation shortly after its release (Hou et al., 2025; Temsah et al., 2025). However, early adoption does not necessarily mean that students will continue using the tool. For medical education, this distinction matters because sustained use may affect curriculum planning, platform governance, and the safety of clinical training.

Research on large language models in medical education has grown rapidly since ChatGPT became widely available. Most existing studies have focused on initial acceptance or performance evaluation, such as whether these models can pass licensing examinations, generate clinically plausible vignettes, or provide responses comparable to those of specialists in standardized scenarios (Ayers et al., 2023; Gilson et al., 2023; Tordjman et al., 2025). Less attention has been paid to continuance, that is, why users keep using a technology after the initial stage of adoption (Bhattacherjee, 2001). For domestically developed Chinese models such as DeepSeek, this question carries additional weight: their data governance frameworks, Chinese-language capabilities, and institutional deployment contexts differ substantially from those of Western tools (Alkhaaldi et al., 2023; Temsah et al., 2025). Recent evidence links social influence and performance expectancy to initial DeepSeek adoption, but what sustains use beyond that stage remains largely unexamined (Al-Maroof et al., 2026).

Continuance intention (CUI) differs from initial adoption in both theory and practice. After users have gained direct experience with a technology, their continued use is shaped less by expectations formed before use and more by judgments formed during use. These judgments include perceived usefulness, ease of interaction, and the extent to which the tool meets prior expectations (Bhattacherjee, 2001; Jung and Jo, 2025). They may also be influenced by system quality, information quality, the fit between the technology and medical learning tasks, and the social environment in which use occurs (Goodhue and Thompson, 1995; Venkatesh and Davis, 2000; DeLone and McLean, 2003). Privacy concerns may further affect students' willingness to rely on AI tools. In healthcare-related learning contexts, concerns about data disclosure may discourage adoption of AI-based tools (Featherman and Pavlou, 2003). Whether privacy concerns persist after routine adoption, or whether they weaken as habit and perceived usefulness accumulate, has not been examined in this context.

We address this gap by asking how repeated experience with DeepSeek produces the cognitive evaluations, satisfaction, and task-fit judgments that drive continued behavioral engagement (CUB), rather than treating post-adoption use as a simple extension of first-use acceptance. An explanatory sequential mixed-methods design was used: structural equation modeling (SEM) tested the relationships among cognitive appraisal, satisfaction, task fit, and continuance behavior, while semi-structured interviews explained unexpected path directions and nonsignificant effects that the structural model alone could not resolve.

## 2 Literature review and research hypotheses

### 2.1 DeepSeek in the medical education context

DeepSeek is relevant to medical education for several reasons. Its reasoning-oriented response style can support case analysis and diagnostic thinking by helping students follow the steps behind a suggested answer, something that conventional search engines do not usually provide (Moëll et al., 2025). It can also generate medical multiple-choice questions, which may support scalable and individualized assessment based on students' knowledge gaps (Hou et al., 2025). In addition, its performance on standardized examinations such as the United States Medical Licensing Examination (USMLE) offers a benchmark for comparison with other large language models, with DeepSeek-V3 and R1 showing results comparable to, and in some cases better than, established models (Tordjman et al., 2025).

These applications, however, come with documented limitations. Hallucinated references, uneven knowledge coverage, and data security concerns remain relevant in clinical training contexts, where students may handle sensitive or professionally consequential information (Temsah et al., 2025). For this reason, DeepSeek cannot be treated as a source that students can use without verification. The more important question, therefore, is why students continue to use the tool despite these limitations.

### 2.2 Cognitive appraisals of continued use

Continued use of an information system is shaped first by how users cognitively appraise it during actual experience, particularly in terms of perceived usefulness (PU) and perceived ease of use (PEOU) (Davis, 1989; Bhattacherjee, 2001). In this study, perceived usefulness refers to whether students believe DeepSeek improves their learning outcomes, while perceived ease of use refers to whether they find the system easy to operate. Both judgments contribute to satisfaction (SAT), which in turn shapes whether students commit to continued use (Deng et al., 2023; Jung and Jo, 2025).

The relationship between perceived ease of use and perceived usefulness is well established. When a tool is easy to navigate, students spend less effort managing the system itself and can devote more attention to learning tasks, which may increase their perception of the tool's usefulness (Ma, 2025). In medical education, perceived usefulness is likely to carry more weight in satisfaction formation than in general consumer contexts. Because medical students often use these tools for academic or clinically relevant tasks, ease of use alone may not be enough to produce satisfaction. Students are more likely to be satisfied when the tool helps them produce better learning outcomes or higher-quality work (Nuryakin et al., 2023).

H1: PU positively predicts SAT.
H2: PEOU positively predicts PU.
H3: PEOU positively predicts SAT.
H4: SAT positively predicts CUI.
H5: CUI positively predicts CUB.

### 2.3 Social influence

Social endorsement from instructors and peers can also shape cognitive appraisals during continued use, particularly by influencing students' perceptions of a tool's usefulness and ease of interaction (Venkatesh and Davis, 2000). In the Unified Theory of Acceptance and Use of Technology (UTAUT) and extended Technology Acceptance Model (TAM) models, subjective norm (SN) has often been linked to perceived usefulness and perceived ease of use because social endorsement can reduce uncertainty about whether a tool is worth using (Venkatesh and Davis, 2000).

Its relationship with satisfaction is less predictable. Strong endorsement from teachers or peers may raise students' expectations before use. If actual performance meets those expectations, satisfaction may increase. If the tool performs below those socially formed expectations, satisfaction may decline because of negative disconfirmation (Oliver, 1980). For this reason, we examine the effects of subjective norm on perceived usefulness, perceived ease of use, and satisfaction separately, without assuming that the path to satisfaction must be positive.

H6: SN positively predicts PU.
H7: SN positively predicts PEOU.
H8: SN predicts SAT.

### 2.4 System quality and information quality

Cognitive appraisals of ease of use are not formed by user psychology alone but are also influenced by the quality of the system itself and the accuracy of the information it produces (DeLone and McLean, 2003). In the context of large language models, system quality (SQ) includes response speed, interface clarity, and stability during use. Information quality (IQ) refers to the accuracy, completeness, and clinical relevance of generated content.

Both dimensions may influence perceived ease of use in online learning environments. A stable and clearly organized system reduces operational difficulty, while accurate and well-structured information lowers the effort required to check and revise generated responses (Machdar, 2019; Al-Fraihat et al., 2020; Widyaningrum et al., 2024). For medical students working under time pressure on academically or professionally consequential tasks, both dimensions carry direct practical weight.

H9: SQ positively predicts PEOU.
H10: IQ has a significant effect on PEOU.

### 2.5 Task-technology fit

Beyond general appraisals of ease and utility, continued behavioral engagement may depend on whether students perceive the technology as fitting the specific demands of their academic tasks (Goodhue and Thompson, 1995). This issue is especially relevant in medical education, where students may use DeepSeek for different purposes, including quick information retrieval, exam preparation, case interpretation, and multi-step clinical reasoning. These tasks differ in their requirements for accuracy, speed, reasoning depth, and domain-specific knowledge.

Task-technology fit (TTF) is shaped by both technology characteristics (TEC) and task characteristics (TAC). Technology characteristics include interface design, linguistic precision, and reasoning depth. Task characteristics include accuracy demands, time pressure, and domain specificity. When students perceive a strong match between DeepSeek's capabilities and their learning tasks, they are more likely to see the tool as useful for continued learning. Prior studies have also linked task-technology fit to continuance intention and satisfaction, especially when the tool consistently turns task inputs into usable outputs (Alyoussef, 2023; Ayyoub et al., 2023).

H11: TEC positively predicts TTF.
H12: TAC positively predicts TTF.
H13: TTF positively predicts CUI.

### 2.6 Expectation confirmation

Because continuance follows repeated use rather than initial adoption alone, students' cognitive appraisals are also updated through expectation confirmation, whereby actual experience is compared with prior expectations (Oliver, 1980; Bhattacherjee, 2001). When the tool meets students' expectations, they are more likely to view it as useful and to feel satisfied with their experience.

When expectation confirmation and perceived usefulness are included in the same model, the direct path from confirmation to satisfaction may become weaker or nonsignificant. One reason is that perceived usefulness may absorb part of the performance evaluation generated by confirmed expectations. Similar mediation patterns have been reported in studies based on the Expectation-Confirmation Model (ECM) (Jung and Jo, 2025; Li et al., 2025). Both paths are therefore included to examine whether this relationship appears in the present sample.

H14: CON positively predicts PU.
H15: CON positively predicts SAT.

### 2.7 Privacy concerns

Beyond cognitive and affective appraisals, continued use may also be constrained by contextual risk perceptions; privacy concerns have been shown to reduce users' willingness to engage with digital services even when those services are perceived as useful (Featherman and Pavlou, 2003). This construct differs from the cognitive appraisal variables discussed above. Rather than reflecting students' judgments about the tool's usefulness or ease of use, it captures a perceived risk that may limit continued engagement even when the tool is seen as functionally valuable (Featherman and Pavlou, 2003).

Privacy concerns may be especially relevant in healthcare-related learning contexts because students may use AI tools when working with clinical cases, patient scenarios, or personally sensitive information. Prior research has shown that privacy concerns can reduce both initial adoption and continued use of AI tools in health-related settings (Featherman and Pavlou, 2003; Zhang et al., 2024). Whether this inhibitory effect persists after routine use develops, or whether it attenuates as habit and perceived usefulness accumulate, remains an open question in post-adoption contexts.

H16: PC negatively predicts CUI.

### 2.8 Psychological focus of the proposed model

The present study is centered on a psychological question: how medical students translate repeated experiences with DeepSeek into continued behavioral engagement. To address this question, the proposed model follows a cognitive affective behavioral logic that places students' post-adoption responses at the center of the analysis. Perceived usefulness, perceived ease of use, and task-technology fit represent student's' cognitive evaluations of the system and its suitability for learning tasks. Satisfaction represents the affective response that emerges from these evaluations during continued use. Continuance intention and continued use behavior reflect the behavioral expression of this process. Other constructs, including system quality, information quality, expectation confirmation, subjective norm, and privacy concerns, are included because they help explain how these core psychological responses are formed or constrained in medical learning contexts.

TAM provides the main cognitive pathway through perceived usefulness and perceived ease of use. These two constructs explain how students judge whether DeepSeek is valuable for learning and whether it is easy to operate during repeated use (Davis, 1989). However, TAM gives less attention to how these judgments are shaped by prior expectations and revised through experience. ECM addresses this post-adoption process by treating expectation confirmation as a source of updated perceived usefulness and satisfaction (Bhattacherjee, 2001). Together, TAM and ECM explain how students' experience with DeepSeek is translated into satisfaction and continuance intention.

The Information Systems Success Model (ISSM) adds the platform-level conditions that influence students' evaluations. System quality and information quality are properties of the tool rather than psychological responses from users. In the context of large language models, these properties are important because output reliability, interface clarity, response stability, and content relevance may vary across tasks and use situations (DeLone and McLean, 2003). Including these constructs allows the model to examine which aspects of DeepSeek's design and output quality are associated with ease of use and satisfaction.

TTF focuses on the alignment between tool capability and task requirements (Goodhue and Thompson, 1995). Medical learning tasks differ considerably, ranging from quick information retrieval to exam preparation, case interpretation, and multi-step clinical reasoning. A tool that works well for one type of task may not be equally suitable for another. TTF captures this perceived alignment and allows its relationship with continuance intention to be examined separately from satisfaction (Alyoussef, 2023; Ayyoub et al., 2023).

Subjective norm and privacy concerns extend the model beyond individual cognition. Peer and instructor endorsement may shape students' perceptions of usefulness and ease of use before and during actual use (Venkatesh and Davis, 2000). Privacy concerns may work in the opposite direction by discouraging continued use even when students evaluate the tool positively (Featherman and Pavlou, 2003). Both factors are relevant in Chinese medical education, where AI tools are increasingly visible in institutional settings and where data sensitivity remains important in clinically oriented learning.

**Figure 1:**
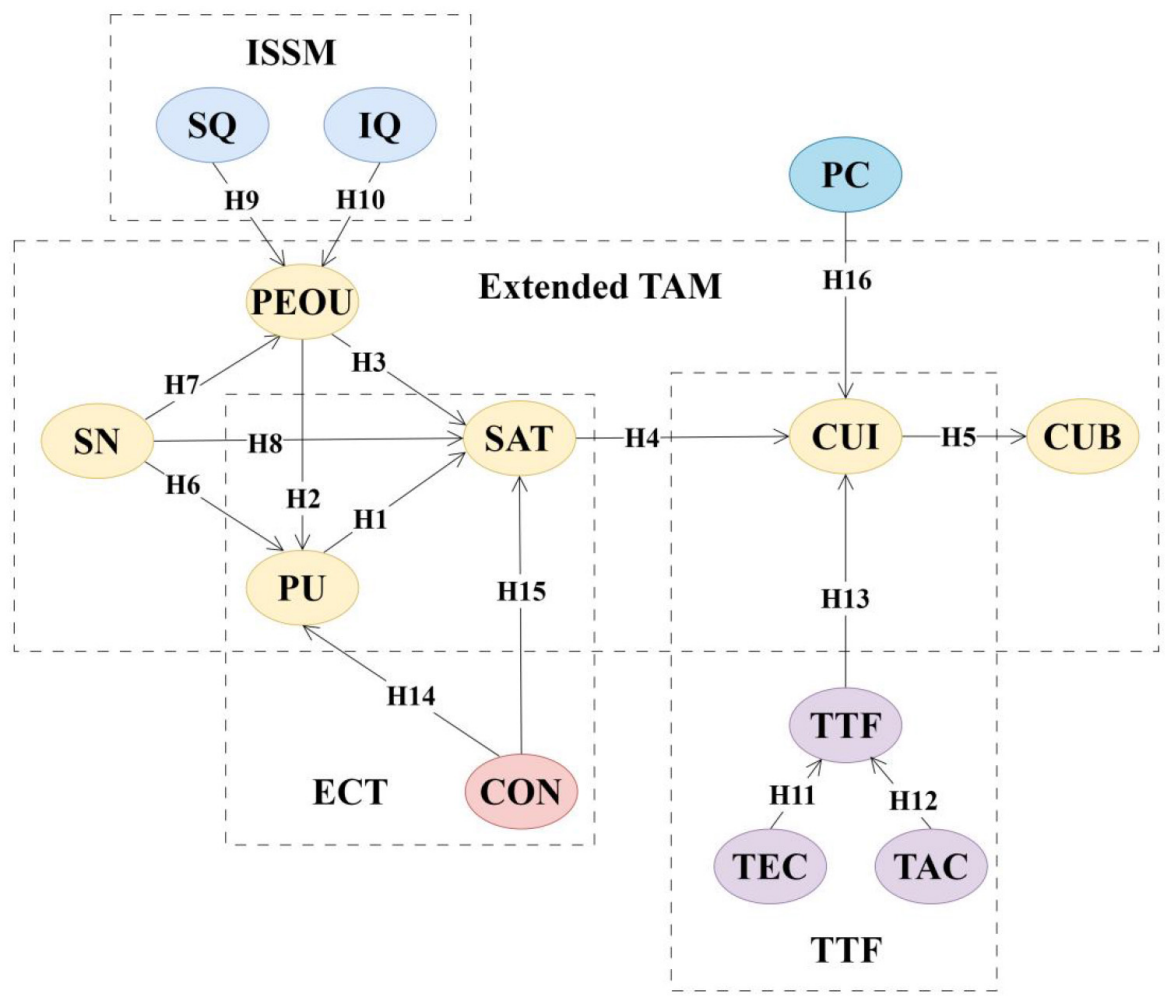
Integrated hypothetical model of Generative AI continuance among medical students.

The integrated framework therefore links four parts of the continuance process: cognitive evaluation, expectation confirmation, platform quality, and task and contextual fit. Figure 1 presents the proposed model.

## 3 Methods

### 3.1 Design

We used an explanatory sequential mixed-methods design. Phase 1 involved a quantitative survey analyzed with SEM to identify predictors of continuance intention. Phase 2 used semi-structured interviews to examine patterns from the quantitative results, including unexpected path directions and nonsignificant relationships. The interviews were designed to clarify these results rather than to replicate the survey findings.

### 3.2 Participants and procedure

#### 3.2.1 Phase 1

Online questionnaires were distributed via the SoJump platform to medical students across multiple Chinese provinces. Responses were screened for completeness and answer quality, yielding 630 valid cases for analysis. This sample size satisfies the >10:1 indicator-to-parameter ratio recommended for SEM (Hair et al., 2019).

#### 3.2.2 Phase 2

Following quantitative analysis, 15 medical students were purposively selected to ensure variation in academic year, major, and self-reported level of DeepSeek use. Interviews were conducted individually face-to-face, each lasting 10–20 min, and were audio-recorded with participants' informed consent. Data collection continued until theoretical saturation was reached. The semi-structured interview guide covered participants' motivations for continued use, perceptions of system performance, task fit, and privacy concerns (see Appendix B).

**Figure 2:**
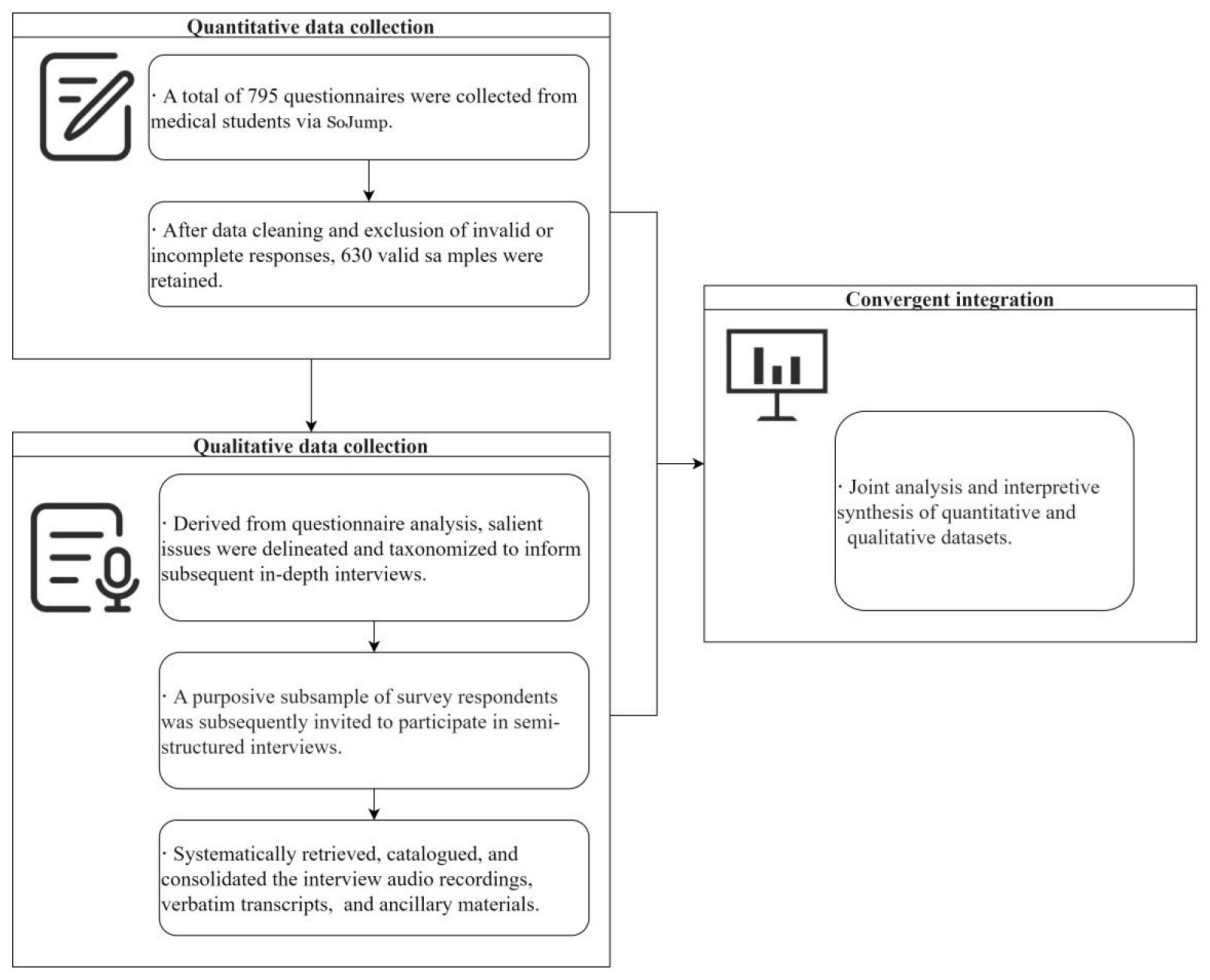
Flowchart of the explanatory sequential mixed-methods design.

Ethics approval was granted by the institutional ethics committee (Approval No.: 2025-008). All procedures adhered to the 1964 Declaration of Helsinki. Written informed consent was obtained from all participants prior to data collection. The study design is illustrated in the Figure 2.

### 3.3 Measures

The questionnaire included 51 items covering 13 latent variables. Responses were recorded on a five-point Likert scale, from 1, strongly disagree, to 5, strongly agree. Items were adapted from established instruments and reviewed by two domain experts for wording clarity and content relevance in the medical education context. The full item wording and scale sources are reported in Appendix A.

### 3.4 Quantitative analysis

SPSS 27.0 was used for descriptive statistics and one-way ANOVA. AMOS 28.0 was used for confirmatory factor analysis (CFA) and structural equation modeling with maximum likelihood estimation.

For the measurement model, factor loadings, composite reliability, average variance extracted (AVE), and Cronbach's alpha were examined. Discriminant validity was assessed using the Fornell–Larcker criterion by comparing the square root of AVE with inter-construct correlations.

To address potential common method bias, two diagnostic checks were performed. Harman's single-factor test was conducted by entering all items into an unrotated principal component analysis to assess whether a single dominant factor emerged (Podsakoff et al., 2003). In addition, variance inflation factors (VIF) were computed for all 13 constructs to detect multicollinearity as a proxy indicator of common method variance (Richardson et al., 2009).

For the structural model, standardized path coefficients and significance levels were estimated. Model fit was assessed using CMIN/df below 3.0, CFI, TLI, and IFI above.90, and RMSEA below.08 (Hair et al., 2019). Indirect effects were examined using the total, direct, and indirect effects matrices in AMOS.

### 3.5 Qualitative analysis

Interview transcripts were analyzed using thematic analysis (Braun and Clarke, 2006). First, transcripts were read and coded inductively to identify recurring ideas in the data. Second, related codes were grouped into subcategories and linked to the relevant theoretical constructs. Third, broader themes were developed and compared with the quantitative results to help interpret patterns that could not be fully explained by SEM alone.

To improve coding credibility, a second researcher reviewed the coding scheme and theme definitions. Disagreements were discussed until agreement was reached. When disagreement remained, a third researcher was consulted. Representative interview quotations for each theme are provided in Appendix C.

## 4 Results

### 4.1 Participant characteristics

**Figure 3:**
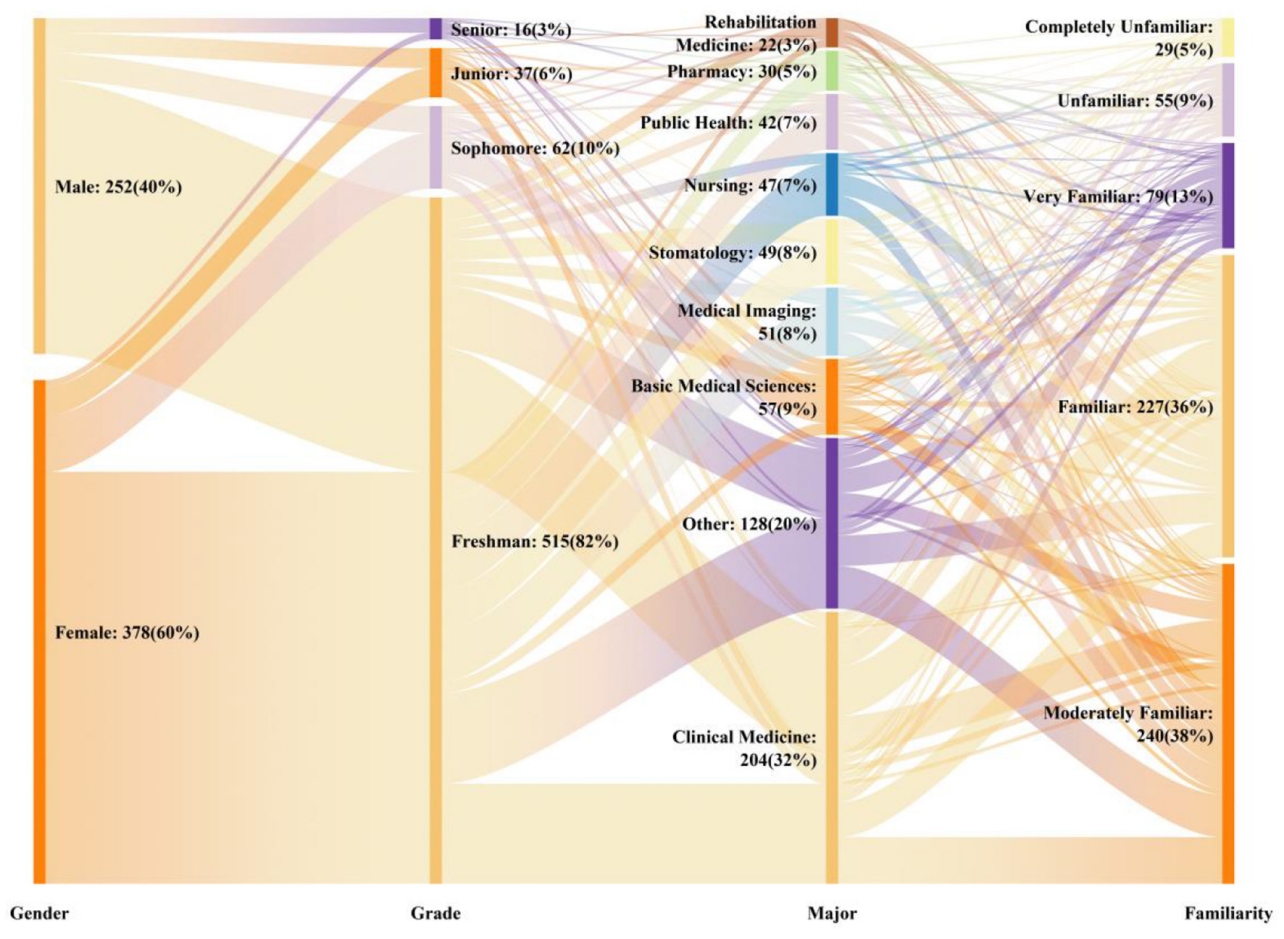
Questionnaire demographic characteristic map.

A total of 630 valid responses were collected. Demographic and background characteristics of the participants are summarized in Figure 3.

In terms of gender, the sample comprised 378 females (60.0%) and 252 males (40.0%). Regarding academic year, the majority were freshmen (*n* = 515, 81.7%), followed by sophomores (*n* = 62, 9.8%), juniors (*n* = 37, 5.9%), and seniors (*n* = 16, 2.5%). Across disciplines, Clinical Medicine represented the largest group (*n* = 204, 32.4%), followed by other fields (*n* = 128, 20.3%) and Basic Medical Sciences (*n* = 57, 9.0%), with the remaining participants distributed across Nursing, Stomatology, Public Health, Medical Imaging, Pharmacy, and Rehabilitation Medicine.

Regarding familiarity with DeepSeek, the majority of participants reported moderate or higher levels of familiarity: moderately familiar (38.1%), Familiar (36.0%), and very familiar (12.5%), while 8.7% were unfamiliar and 4.6% completely unfamiliar. Overall, approximately 86.6% of respondents had some degree of familiarity with DeepSeek prior to the study.

### 4.2 Common method bias (CMB) assessment

Harman's single-factor test showed that the first unrotated factor explained 43.1% of the total variance, slightly above the commonly referenced 40% threshold (Podsakoff et al., 2003). Given the limitations of Harman's test as a standalone diagnostic, this result was interpreted with caution (Richardson et al., 2009). VIF values ranged from 1.165 for PC to 4.715 for PU, below the threshold of 5, indicating no serious multicollinearity. Common method bias was not considered a major threat to the structural estimates, but the Harman test result was considered in the interpretation.

### 4.3 Measurement model

Factor loadings for the 51 indicators ranged from 0.643 to 0.822, and all were significant at *p* < 0.001. Composite reliability exceeded 0.70 for all constructs. AVE values ranged from 0.527 to 0.638, indicating satisfactory convergent validity. Discriminant validity was assessed using the Fornell–Larcker criterion, as shown in Table 1. Full CFA results are reported in Table 2.

**Table 1:** Discriminant validity: Fornell–Larcker criterion matrix.

Construct	SQ	IQ	PC	CUB	CUI	TTF	TAC	TEC	CON	SAT	PEOU	SN	PU
SQ	0.746												
IQ	0.867	0.741											
PC	0.279	0.178	0.799										
CUB	0.621	0.639	0.215	0.757									
CUI	0.800	0.679	0.330	0.632	0.788								
TTF	0.869	0.775	0.311	0.572	0.801	0.78							
TAC	0.823	0.745	0.305	0.614	0.782	0.901	0.739						
TEC	0.841	0.758	0.276	0.556	0.733	0.906	0.866	0.741					
CON	0.723	0.640	0.274	0.552	0.711	0.827	0.814	0.864	0.777				
SAT	0.846	0.758	0.295	0.627	0.922	0.826	0.807	0.781	0.715	0.768			
PEOU	0.879	0.724	0.383	0.554	0.880	0.798	0.820	0.744	0.722	0.940	0.726		
SN	0.831	0.740	0.359	0.546	0.783	0.783	0.761	0.734	0.655	0.798	0.861	0.729	
PU	0.879	0.754	0.35	0.598	0.891	0.875	0.845	0.81	0.731	0.978	0.903	0.873	0.769

Values on the diagonal are square roots of AVE.

**Table 2:** Confirmatory factor analysis results.

Facet	Item	Parameter estimates				Factor loading	Convergent validity	Composite reliability	Cronbach's α
		Unsth	S.E.	C.R.	*P*	Std	AVE	CR	
SQ	SQ1	1				0.717	0.557	0.790	0.785
	SQ2	1.060	0.057	18.757	^***^	0.782			
	SQ3	0.999	0.056	17.718	^***^	0.738			
IQ	IQ1	1				0.732	0.549	0.829	0.829
	IQ2	0.937	0.061	15.356	^***^	0.643			
	IQ3	1.017	0.054	18.733	^***^	0.783			
	IQ4	1.050	0.055	19.015	^***^	0.796			
PC	PC1	1				0.781	0.638	0.876	0.875
	PC2	0.984	0.049	19.980	^***^	0.784			
	PC3	1.063	0.051	20.997	^***^	0.822			
	PC4	1.038	0.050	20.640	^***^	0.808			
CUB	CUB1	1				0.697	0.574	0.842	0.845
	CUB2	1.118	0.063	17.864	^***^	0.811			
	CUB3	1.003	0.064	15.599	^***^	0.692			
	CUB4	1.053	0.058	18.014	^***^	0.820			
CUI	CUI1	1				0.797	0.621	0.868	0.867
	CUI2	1.085	0.048	22.383	^***^	0.807			
	CUI3	1.033	0.051	20.341	^***^	0.750			
	CUI4	1.079	0.049	22.030	^***^	0.798			
TTF	TTF1	1				0.788	0.608	0.861	0.860
	TTF2	1.043	0.048	21.908	^***^	0.798			
	TTF3	0.979	0.047	20.756	^***^	0.764			
	TTF4	1.022	0.049	20.880	^***^	0.768			
TAC	TAC1	1				0.768	0.545	0.827	0.826
	TAC2	0.990	0.053	18.649	^***^	0.729			
	TAC3	1.016	0.054	18.965	^***^	0.739			
	TAC4	1.022	0.056	18.315	^***^	0.717			
TEC	TEC1	1				0.775	0.548	0.829	0.831
	TEC2	1.027	0.052	19.799	^***^	0.756			
	TEC3	0.943	0.051	18.652	^***^	0.719			
	TEC4	0.951	0.052	18.378	^***^	0.710			
CON	CON1	1				0.812	0.603	0.859	0.857
	CON2	0.962	0.049	19.790	^***^	0.734			
	CON3	0.936	0.045	20.886	^***^	0.766			
	CON4	0.967	0.044	21.842	^***^	0.793			
SAT	SAT1	1				0.787	0.59	0.852	0.851
	SAT2	0.901	0.045	20.090	^***^	0.737			
	SAT3	0.990	0.047	21.298	^***^	0.772			
	SAT4	1.026	0.048	21.437	^***^	0.776			
PEOU	PEOU1	1				0.754	0.527	0.816	0.817
	PEOU2	1.003	0.055	18.380	^***^	0.719			
	PEOU3	1.019	0.055	18.417	^***^	0.72			
	PEOU4	1.004	0.055	18.090	^***^	0.709			
SN	SN1	1				0.724	0.531	0.819	0.819
	SN2	1.042	0.057	18.137	^***^	0.768			
	SN3	0.986	0.060	16.565	^***^	0.700			
	SN4	0.998	0.058	17.067	^***^	0.721			
PU	PU1	1				0.783	0.591	0.852	0.85
	PU2	1.041	0.048	21.758	^***^	0.787			
	PU3	1.037	0.047	22.015	^***^	0.794			
	PU4	1.001	0.052	19.113	^***^	0.709			

^***^p < 0.001.

### 4.4 Structural model fit

The structural model showed an acceptable fit to the data. The main fit indices met commonly used criteria, with χ^2^/df = 2.521, RMSEA = 0.049, RMR = 0.030, CFI = 0.914, TLI = 0.908, and IFI = 0.915. GFI = 0.832, and AGFI = 0.812 were lower than the 0.90 criterion but remained within a range often considered acceptable in complex models. The parsimony-adjusted indices were PNFI = 0.806 and PCFI = 0.815. Model fit was therefore adequate for path analysis.

### 4.5 Hypothesis testing

Table 3 reports the structural path estimates and hypothesis testing results. Fourteen of the 16 hypothesized paths reached statistical significance. SQ positively predicted PEOU, and SN positively predicted both PEOU and PU. PEOU was positively associated with PU and SAT, while PU had the strongest direct effect on SAT. SAT further predicted CUI, and CUI predicted CUB.

**Table 3:** Structural path estimates.

Path	Estimate	S.E.	C.R.	*P*	Result
SQ → PEOU	0.898	0.160	5.596	^***^	Supported
IQ → PEOU	−0.247	0.095	−2.612	0.009	Supported
SN → PEOU	0.322	0.087	3.691	^***^	Supported
SN → PU	0.239	0.079	3.017	0.003	Supported
PEOU → PU	0.673	0.091	7.384	^***^	Supported
CON → PU	0.116	0.034	3.411	^***^	Supported
SN → SAT	−0.259	0.091	−2.841	0.004	Supported
PU → SAT	0.907	0.192	4.733	^***^	Supported
PEOU → SAT	0.368	0.161	2.283	0.022	Supported
CON → SAT	0.005	0.039	0.137	0.891	Not supported
TEC → TTF	0.486	0.079	6.141	^***^	Supported
TAC → TTF	0.508	0.079	6.438	^***^	Supported
SAT → CUI	0.769	0.057	13.600	^***^	Supported
TTF → CUI	0.157	0.047	3.299	^***^	Supported
PC → CUI	0.031	0.024	1.316	0.188	Not supported
CUI → CUB	0.732	0.056	13.031	^***^	Supported

^***^p < 0.001, Bootstrap resampling (2,000 samples, bias-corrected) confirmed the significance of all supported hypotheses.

In the task-technology fit pathway, TEC and TAC both positively predicted TTF. TTF also had a significant positive effect on CUI, although this effect was weaker than the effect of SAT. Two paths were not supported: CON did not significantly predict SAT, and PC did not significantly predict CUI. Two significant paths also showed unexpected negative directions: IQ to PEOU and SN to SAT. These patterns were examined further in the qualitative phase.

### 4.6 Semi-structured interview results

#### 4.6.1 Analytical approach

Fifteen semi-structured interview transcripts were analyzed using qualitative content analysis. Coding followed three steps: open coding, axial coding, and selective coding. Initial codes were generated from participants' statements, grouped into subcategories, and then linked to the 13 theoretical constructs in the integrated TAM-TTF framework. These constructs were further organized into four themes: adoption drivers, system performance and fit, risk perceptions, and user outcomes. Coding continued until no new subcategories appeared after the 13th transcript. Full coding results are provided in Appendix C.

#### 4.6.2 Theme 1: adoption drivers

Social influence mainly shaped initial adoption. Several participants tried DeepSeek because it was widely discussed among classmates or online communities. One participant stated, “Because everyone around me was using it, I decided to try it myself” (P3). Another mentioned online promotion: “I saw online promotions describing DeepSeek as China's AI assistant, so I gave it a try” (P12).

Perceived ease of use was the most frequently mentioned factor. Students described DeepSeek as easy to access and simple to operate. Typical comments included, “You can use it simply by entering text” (P3), and “No VPN is required; it can be accessed at any time” (P12). Perceived usefulness appeared in literature retrieval, academic writing, and exam preparation. For example, one participant noted that DeepSeek “provides paper frameworks and ideas, helping to organize my thinking” (P10).

#### 4.6.3 Theme 2: system performance and fit

Participants reported both strengths and weaknesses in system performance. System quality concerns focused on lag, unstable access, and “system busy” prompts. One participant said, “It sometimes lags, displaying a ‘system busy' prompt” (P3). Another noted that the system “frequently enters a busy state, which negatively affects the experience” (P10).

Information quality was another concern. Participants mentioned false information, formatting problems, and overly extended answers. Two participants referred to the same case in which DeepSeek generated a false postgraduate entrance score, describing it as “highly misleading” (P9, P10). At the same time, students valued DeepSeek's Chinese-language fluency and contextual understanding. One participant said, “It understands the meaning of questions and provides deeper, more comprehensive answers” (P7).

Task-technology fit was most visible in specialized academic tasks. Participants found DeepSeek useful for literature retrieval, postgraduate application searches, and clinical exam preparation. One participant stated, “In highly specialized fields, retrieval is faster and it is easier to find the content I need” (P3).

#### 4.6.4 Theme 3: risk perceptions

Risk perceptions were mainly related to future pricing and data security. Several participants said their continued use depended on whether DeepSeek remained free or reasonably priced. One participant stated, “I worry that it will become paid in the future” (P1). Data security was also mentioned, especially when the tool might be used with clinical cases or sensitive information. These concerns did not stop most participants from using DeepSeek, but they made students more cautious about relying on it.

#### 4.6.5 Theme 4: user outcomes

Overall satisfaction was high, although participants also noted problems such as server instability and limited output formats. One participant said, “I am very satisfied” (P1), while another gave DeepSeek a high rating but deducted points because of unstable access and limited output formats (P11).

Continuance intention was common but conditional. Many students were willing to continue using DeepSeek while it remained free. One participant explained, “It is currently free, so the usage cost is low” (P6). Some participants were willing to consider payment if the price was reasonable, while others said they would switch to a better tool if one became available. Actual continued use was mainly task-driven, especially for academic writing, exam preparation, and postgraduate information searches.

Across all four themes, students continued using DeepSeek primarily because access was free, operation was simple, and the tool handled common academic tasks adequately. Their reservations centered on server instability, inaccurate outputs, potential fees, and data security.

## 5 Discussion

The continued use of DeepSeek by medical students cannot be reduced to a single motivational force. The results reveal a layered process in which cognitive evaluations of system attributes feed into an affective state of satisfaction, which then activates behavioral intention and subsequently actual use. Rather than treating each path coefficient in isolation, this discussion organizes the findings around a cognitive affective behavioral hierarchy, allowing the psychological mechanisms underlying continuance to be examined at three distinct levels of abstraction.

### 5.1 The cognitive layer: how students appraise system attributes

At the cognitive layer, students evaluated what DeepSeek could offer and how much effort it required to use. PEOU predicted PU: when students found DeepSeek easy to navigate, they redirected attention from operational management to learning tasks, which raised their perception of the tool's usefulness (Davis, 1989; Bhattacherjee, 2001). Three system-level constructs were linked to PEOU in different ways. System quality showed the strongest effect on PEOU, confirming that stable response speed and interface clarity directly lower the effort students associate with operating the tool (DeLone and McLean, 2003). Information quality had a significant negative effect on PEOU. When content accuracy is inconsistent, students need to re-query, cross-check, or consult other sources. These extra verification steps increase the practical effort of using the tool (Al-Fraihat et al., 2020).

Benchmark data corroborate this pattern: DeepSeek produces hallucinations even in high-accuracy clinical scenarios (Sandmann et al., 2025), making output unreliability a platform-level problem rather than a marginal user concern. The interview data add a more concrete explanation. Two participants independently mentioned the same fabricated postgraduate entrance score and described how that incident changed their later verification routines (P9, P10). In medical education, even a single memorable error may carry disproportionate weight because students are working with academically and professionally consequential information. For platform developers, reducing output inaccuracy is not a cosmetic concern; it directly determines how much verification work students must absorb during routine use.

The TTF pathway operated through a parallel cognitive route. Technology characteristics and task characteristics both significantly predicted TTF, and TTF then predicted continued usage intention. TTF reflects whether students believe DeepSeek fits the demands of their specific learning tasks (Goodhue and Thompson, 1995). Its effect on continued usage intention was independent of the satisfaction pathway, meaning that students may continue using DeepSeek because it fits certain tasks well, even when their overall satisfaction is limited.

This task-fit effect is especially relevant for students with specialized academic needs, such as pharmaceutical literature retrieval or postgraduate application searches. In these contexts, DeepSeek may remain useful despite instability or occasional inaccuracy if it helps students' complete tasks more efficiently than available alternatives. Al-Maroof et al. (2026) similarly found that performance cognitions predicted usage intention independently of attitudinal measures, which is consistent with the independent TTF path observed here (Al-Maroof et al., 2026).

At the cognitive layer, system quality and information quality had opposite effects on PEOU: stable performance lowered operational effort, while inconsistent accuracy raised it by adding verification steps. A single memorable error, such as the fabricated postgraduate entrance score recalled by two participants (P9, P10), may carry disproportionate weight because students working with academically consequential content are especially sensitive to credibility failures.

### 5.2 The affective layer: how cognitive appraisals become satisfaction

Expectation confirmation significantly predicted perceived usefulness but did not significantly predict satisfaction. This differs from Bhattacherjee's original ECM, where confirmation is expected to influence satisfaction more directly (Bhattacherjee, 2001). One possible explanation is that confirmed expectations first update students' usefulness judgments, while satisfaction is affected by other experiences occurring at the same time. Server lag, pricing anxiety, formatting problems, and output inaccuracies may weaken the emotional effect of confirmed expectations. Students may therefore recognize that DeepSeek is useful without becoming more satisfied with the overall experience. This attenuation of the CON to SAT path when PU is present has been observed in other educational AI continuance studies (Jung and Jo, 2025; Li et al., 2025), suggesting that usefulness judgments absorb much of the motivational content of confirmed expectations.

SAT predicted CUI more strongly than any other construct in the model, confirming satisfaction as the primary gateway between evaluation and behavior. Both PEOU and PU significantly predicted satisfaction, showing that ease of interaction and perceived usefulness matter not only as cognitive judgments but also as parts of the user experience.

PU contributed more strongly to SAT than PEOU. For medical students whose tasks carry academic or clinical stakes, output quality outweighs operational convenience as a satisfaction driver: ease of use lowers the entry cost, but usefulness determines whether repeated use feels worthwhile.

The negative path from social influence to satisfaction deserves attention. Social influence positively predicted both PEOU and PU, which means that peer and instructor endorsement helped students recognize DeepSeek's functions (Venkatesh and Davis, 2000). At the same time, endorsement may have raised expectations before use. When students later encountered server instability, inaccurate content, or poorly formatted responses, satisfaction may have declined because actual performance did not match the expectations created by social promotion. Expectation-disconfirmation theory predicts this pattern (Oliver, 1980): strong promotional endorsement inflates pre-use expectations, and subsequent performance failures such as server lag, inaccurate content, and formatting errors produce negative disconfirmation that depresses satisfaction below its baseline level.

The nonsignificant PC path should not be read as evidence that privacy was unimportant to students. Risk concerns may remain inactive until a specific trigger appears, such as a pricing change, data leak, or misuse of information (Featherman and Pavlou, 2003). The free-access period during data collection may have suppressed behavioral responses to these concerns, as pricing changes or data incidents had not yet occurred for most participants.

The interviews showed that risk concerns were still present. Many participants linked continued use to whether DeepSeek would remain free, and pricing anxiety appeared frequently in the qualitative data. The difference between the survey result and the interview data may reflect a limitation of standard Likert-scale privacy items in a free-access context. Longitudinal data collected after pricing changes or platform policy changes would provide a clearer test of how these concerns affect continued use.

Across the affective layer, satisfaction responded more to in-use performance failures than to pre-use expectation fulfillment. The nonsignificant CON to SAT path and the negative SN to SAT coefficient together indicate that inflated expectations and disruptive experiences are the primary suppressors of satisfaction. This asymmetry is consistent with negativity bias in satisfaction formation (Oliver, 1980): adverse events carry disproportionate weight in users overall affective evaluations.

### 5.3 The behavioral layer: from intention to actual use

Continued usage intention strongly predicted actual use behavior. Actual use, however, was task-concentrated: interview participants described using DeepSeek almost exclusively for academic writing, examination preparation, and postgraduate application searches. Habit formation theory accounts for this distribution: repeated use in a stable task context gradually converts deliberate choices into routine behavior (Limayem et al., 2007).

Curriculum structure may therefore affect continued use. Students whose courses involve frequent writing, literature searching, or examination preparation may develop stronger DeepSeek-use habits than students whose programs rely more on problem-based learning, clinical placement, or face-to-face discussion. The TTF path here operated independently of the satisfaction pathway (β = 0.157), suggesting that task relevance functions as a distinct behavioral driver that does not depend on overall affective appraisal. Continued use of AI tools may depend not only on platform quality, but also on whether students repeatedly encounter tasks for which the tool is useful.

At the behavioral layer, the dual-pathway structure, with satisfaction driving continuance intention while task-technology fit contributes independently, shows that continued use is not a unitary outcome. Students may continue using DeepSeek for functionally specific reasons even when overall satisfaction is constrained by system instability or pricing uncertainty. Satisfaction-focused improvements such as reducing server lag and improving response formatting may not retain students who continue primarily for task-specific reasons. Conversely, expanding task-fit capabilities may not improve overall satisfaction among students whose main concern is reliability. Platform and curriculum interventions need to target both pathways separately.

### 5.4 Where the three layers interact: the SN–SAT chain and its limits

The path from social influence to actual behavior shows how the three layers interact and constrain each other. At the cognitive layer, social influence increased PEOU and PU (Venkatesh and Davis, 2000), reflecting peer and instructor endorsement's role in communicating what the tool can do. At the affective layer, however, the same endorsement reduced satisfaction, likely because strong promotion raised expectations that the tool did not consistently meet (Oliver, 1980). This split, where cognitive facilitation occurs alongside affective suppression, has been documented in cognitive-affective-behavioral analyses of student AI trust, and the SN data here replicate that pattern (Jung and Jo, 2025). Satisfaction acted as the key transition point between evaluation and behavior. Cognitive appraisals such as perceived usefulness and ease of use influenced continuance mainly when they were successfully translated into positive affect. Improving functionality alone is therefore insufficient if students simultaneously experience unstable access, inaccurate outputs, or unmet expectations. Affective responses exerted stronger proximal influence on continued use than cognitive evaluations alone (Al-Maroof et al., 2026), which aligns with the dominant SAT to CUI path over the TTF to CUI path in the present model. Early adoption momentum does not guarantee durable use. Whether current DeepSeek engagement converts into stable behavioral routines depends on consistent accuracy and task-appropriate output, conditions that the present data show remain unmet for a substantial share of students.

Two additional paths clarify boundary conditions of the main model. Expectation confirmation significantly predicted perceived usefulness but not satisfaction: students updated their usefulness judgments when DeepSeek met prior expectations, but those cognitive updates did not convert into greater satisfaction when server instability, inaccurate outputs, and pricing uncertainty were simultaneously present. Satisfaction at the affective layer appeared to depend less on whether the tool met prior expectations and more on whether continued use was free from disruptive failures. Privacy concerns did not significantly predict continuance intention at the time of data collection. The interview data corroborate this: most students named data security as a concern but continued using DeepSeek because it was free and functionally adequate. These concerns appeared dormant rather than absent. Several participants stated that a pricing change (P1, P6), while data security risks were also noted (P9), which indicates that privacy related behavioral effects may emerge under conditions not captured by a single wave survey.

### 5.5 Theoretical contributions

Organizing the findings around a cognitive-affective-behavioral hierarchy explains why significant path coefficients alone produce an incomplete picture: the SAT to CUI coefficient only becomes interpretable once the upstream conditions that shape satisfaction are traced. It links platform quality, students' cognitive evaluations, satisfaction, continuance intention, and actual use behavior in a single explanatory sequence. By integrating TAM, ECM, ISSM, and TTF, the framework shows how system-level conditions, expectation confirmation, task fit, and user appraisals jointly shape continued AI use in medical education.

The negative IQ-PEOU and SN-SAT paths add further theoretical value. In standard adoption models, information quality and social influence are often treated as positive drivers. In this study, their effects were more conditional. Unreliable information increased the effort required to verify outputs, which made the tool feel less easy to use. Strong social endorsement may also have raised students' expectations, making satisfaction harder to maintain when actual performance was unstable. Perceived effort and satisfaction were therefore shaped not only by interface design and functional utility but also by verification burden and expectation inflation, two factors absent from standard adoption models.

The qualitative data further showed that behavioral engagement was task-concentrated rather than platform-general: participants linked continued use to low cost and specific task needs, while actual use clustered around academic writing, examination preparation, and postgraduate information searches. Continuance models of AI use that treat all use instances as equivalent miss this task-level variation, which the present data show is behaviorally consequential.

### 5.6 Practical implications

For platform development, information accuracy deserves priority. The negative relationship between IQ and PEOU indicates that unreliable outputs increase students' verification burden and reduce the perceived ease of use. Improvements in medical-domain accuracy, citation reliability, and output verification may therefore have wider effects than interface optimization alone. Given reported hallucinations in clinical scenarios, medical content fine-tuning and built-in citation checking are particularly important (Sandmann et al., 2025).

For medical education, AI literacy training should be placed within the tasks where students already use DeepSeek, such as academic writing, literature retrieval, and examination preparation. The interview data showed that students were aware of possible inaccuracies, but this awareness did not always lead to systematic checking. Task-based training in critical appraisal could help students turn general caution into concrete verification habits.

For institutional policy, the negative SN-SAT path calls for more careful communication about AI tools. Promotion may increase initial adoption, but it may also create inflated expectations. Universities and departments should present both the strengths and limitations of DeepSeek, especially in relation to content accuracy, system stability, and appropriate use in clinically related learning.

## 6 Limitations and suggestions for future research

Three methodological limitations affect the scope of these conclusions. The cross-sectional design captured students' perceptions and use behavior at a single point in time, so it could not examine how satisfaction, privacy concerns, or continued use may change as students gain longer-term experience with DeepSeek. Given that DeepSeek was still evolving during data collection, platform updates, pricing changes, or data security events occurring after the survey may have already altered the patterns reported here. A longitudinal design, collecting data before and after a pricing change or service disruption, would test whether the currently dormant privacy concerns become behavioral determinants under different platform conditions.

The sample, though drawn from multiple provinces, remains subject to sampling imbalance and self-selection bias: students with stronger AI interest or higher digital literacy were more likely to participate. Students who were more familiar with AI tools or more interested in DeepSeek may have been more likely to participate. Continued use behavior was also measured through self-reported data rather than objective usage logs, which may introduce recall bias or social desirability bias. The qualitative phase was also limited to 15 participants, which restricts the range of experiences represented, particularly for senior students and students in less common specializations. Stratified sampling, larger interview cohorts, and objective usage logs, where ethically and technically feasible, would address the sampling and self-report limitations identified above.

## Data Availability

The original contributions presented in the study are included in the article/Supplementary material, further inquiries can be directed to the corresponding authors.
