# Supplementary material for: Psychological mechanisms underlying medical students' continued use of DeepSeek: an explanatory sequential mixed-methods study
Source: Front Psychol. 2026 Jul 8;17:1875736. doi: 10.3389/fpsyg.2026.1875736 (PMC13390673; doi:10.3389/fpsyg.2026.1875736)
Supplement: Supplementary file 1 [file Supplementary_file_1.docx]

**Appendix A**

| **Questionnaire on the Use of DeepSeek by Medical Students** | | |
| --- | --- | --- |
| Gender | Male |  |
|  | Female |  |
| Academic Year / Grade Level | First Year Undergraduate |  |
|  | Second Year Undergraduate |  |
|  | Third Year Undergraduate |  |
|  | Fourth Year Undergraduate |  |
|  | Fifth Year Undergraduate |  |
|  | Postgraduate and above |  |
| Field of Study / Major | Chinese Medicine |  |
|  | Clinical Medicine |  |
|  | Basic Medical Sciences |  |
|  | Pharmacy |  |
|  | Dentistry / Stomatology |  |
|  | Nursing |  |
|  | Rehabilitation Medicine |  |
|  | Public Health |  |
|  | Preventive Medicine |  |
|  | Medical Imaging / Laboratory Technology |  |
|  | Other (Please specify) |  |
| Overall Familiarity with DeepSeek | Very Familiar |  |
|  | Familiar |  |
|  | Moderately Familiar |  |
|  | Slightly Familiar |  |
|  | Not at all Familiar |  |
| Expectation Confirmation (CON) | 1. My experience with DeepSeek met my expectations. | (Bhattacherjee et al., 2008) |
|  | 2. My experience with DeepSeek exceeded my expectations. |  |
|  | 3. Learning to use DeepSeek was easier than I expected. |  |
|  | 4. The actual process of using DeepSeek was simpler than I had imagined. |  |
| Technology Characteristics (TEC) | 1. It is useful to me that DeepSeek organizes and presents the most relevant content logically, rather than just listing it. | (Zhou et al., 2010) |
|  | 2. I believe DeepSeek can accurately understand my intent based on my descriptions. |  |
|  | 3. DeepSeek provides a rapid service for medical research and learning activities. |  |
|  | 4. DeepSeek provides a secure service for medical research and learning activities. |  |
| Task Characteristics (TAC) | 1. I require the content provided by DeepSeek to be accurate. | (Zhou et al., 2010) |
|  | 2. I often need to acquire information quickly when using DeepSeek. |  |
|  | 3. I need DeepSeek to provide me with more creative ideas. |  |
|  | 4. I frequently need to use DeepSeek to solve problems within my professional field. |  |
| Task-Technology Fit (TTF) | 1. The functionalities of DeepSeek align with my needs. | (Zhou et al., 2010) |
|  | 2. DeepSeek is capable of meeting my academic and professional requirements. |  |
|  | 3. The content provided by DeepSeek is applicable. |  |
|  | 4. For specific tasks, using DeepSeek allows for the rapid acquisition of necessary content. |  |
| Information Quality （IQ） | 1. The information provided by DeepSeek is up-to-date. | (Zhou, 2013) |
|  | 2. The information provided by DeepSeek is accurate. |  |
|  | 3. The information provided by DeepSeek is comprehensive. |  |
|  | 4. DeepSeek ensures that users can access trustworthy content. |  |
| System Quality （SQ） | 1. DeepSeek is reliable. | (Zhou, 2013) |
|  | 2. DeepSeek is effective. |  |
|  | 3. The layout of DeepSeek is clear. |  |
|  |  |  |
| Subjective Norm (SN) | 1. My instructors/professors think it is a good idea for me to use DeepSeek. | (Venkatesh et al., 2003) |
|  | 2. My peers think it is a good idea for me to use DeepSeek. |  |
|  | 3. My parents approve of my use of DeepSeek. |  |
|  | 4. My university or hospital encourages and supports the use of the DeepSeek system. |  |
| Perceived Usefulness (PU) | 1. I believe that using DeepSeek allows me to get answers to my questions. | (Yen et al., 2010) |
|  | 2. Using the DeepSeek system has improved my work efficiency. |  |
|  | 3. Using the DeepSeek system has enhanced the effectiveness of my work. |  |
|  | 4. I find the DeepSeek system to be useful in my medical work. |  |
| Perceived Ease of Use (PEOU) | 1. Interacting with DeepSeek is clear and understandable. | (Yen et al., 2010) |
|  | 2. Using DeepSeek does not require significant mental effort from me. |  |
|  | 3. The cost of using DeepSeek is low. |  |
|  | 4. It is easy to get DeepSeek to do what I want it to do. |  |
| Satisfaction（SAT） | 1. I think using DeepSeek for content generation is a good idea. | Bhattacherjee (2001) |
|  | 2. I believe that using DeepSeek is beneficial to my work. |  |
|  | 3. I find the process of using DeepSeek to be enjoyable. |  |
|  | 4. I like using DeepSeek for content generation. |  |
| Privacy Concerns (PC) | 1. I am concerned that access to my private information by unknown parties could negatively affect my privacy. | (Dinev and Hart, 2006) |
|  | 2. I am concerned that private information within the DeepSeek system could be misused. |  |
|  | 3. I am concerned that unknown parties can access my private information within the DeepSeek system. |  |
|  | 4. I am concerned that inadequate privacy protection measures in the DeepSeek system could lead to information leakage. |  |
| Continuance Intention（CUI） | 1. I intend to continue exploring and learning about DeepSeek. | Bhattacherjee (2001) |
|  | 2. I intend to continue using DeepSeek. |  |
|  | 3. I will recommend DeepSeek to others. |  |
|  | 4. I will continue to proactively use DeepSeek to aid my studies or work. |  |
| Continuous Usage Behavior（CUB） | 1. I have become dependent on DeepSeek in my daily studies or research. | Bhattacherjee et al. (2008) |
|  | 2. When I encounter problems in my studies or research, I will prioritize using DeepSeek for answers. |  |
|  | 3. I would feel inconvenienced if I could not use DeepSeek. |  |
|  | 4. DeepSeek has become an essential tool for my studies and research. |  |

**Appendix B**

| **Semi-Structured Interview Questionnaire** |
| --- |
| Q1.What channels do you typically use to acquire academic materials? |
| Q2.Previously, which tools or platforms did you primarily rely on for literature searches and information retrieval? |
| Q3.What were your initial reasons for choosing DS? |
| Q5.What features or characteristics of this tool make you feel it is more suitable for your needs? |
| Q6. Could you please describe your overall user experience? |
| Q7. Which features have you found to be helpful? |
| Q8. Are the user interface and workflow intuitive? Do you have any suggestions for improvement? |
| Q9. How do you assess the accuracy and credibility of the content generated by the tool? |
| Q10.Have you ever been skeptical of the generated content while using the tool? If so, how did you verify it? |
| Q11.What are the primary factors that motivate you to continue using this tool? |
| Q12.If a new, similar product were to become available, would you consider switching? |
| Q13.Compared to other tools, how do you evaluate its cost-effectiveness? |
| Q14.Does the cost factor influence your decision to choose and use this tool? |
| Q15.As a first-time user, did you encounter any difficulties with its operation? |
| Q16.Compared to tools you have used previously, is the interface and operational design of this tool easier to learn? |
| Q17.What is your current level of satisfaction with the tool? |
| Q18.What is the feature you are most satisfied with? |
| Q19.What is the feature you are least satisfied with? |
| Q20.What new features would you like to see the tool offer for your future studies and research? |
| Q21.What has been your most memorable experience while using the tool? |
| Q22.Is there anything else you would like to add? |

**Appendix C**

| Higher-Order Theme | Main Category | Subcategory | *n* | % | Representative Quotation |
| --- | --- | --- | --- | --- | --- |
| Adoption Drivers | SN (3) | Peer Influence | 1 | 6.70% | *"Because everyone around me was using it, I decided to try it myself."* (P3) |
|  |  | Supervisor Recommendation | 1 | 6.70% | *"I mainly obtain materials through my supervisor and instructors."* (P8) |
|  |  | Online Hype | 1 | 6.70% | *"I saw online promotions describing it as China's AI assistant, so I gave it a try."* (P12) |
|  | PEOU (18) | Simple Interface | 6 | 40% | *"The interface is clean; I can start searching immediately."* (P1) |
|  |  | Zero Learning Cost | 8 | 53% | *"You can use it simply by entering text."* (P3) |
|  |  | Anytime Access | 4 | 27% | *"No VPN is required; it can be accessed at any time."* (P12) |
|  | PU (9) | Efficiency Gain | 4 | 27% | *"It allows me to quickly obtain the information I need and improves research efficiency."* (P3) |
|  |  | Paper Framework | 3 | 20% | *"It provides paper frameworks and ideas, helping to organize my thinking."* (P10) |
|  |  | In-Depth Analysis | 2 | 13% | *"It analyzed grammatical structures in detail, which I had never encountered before."* (P4) |
|  |  | Exam Support | 2 | 13% | *"I rely entirely on it to search for questions during final-exam preparation."* (P9) |
| System Performance & Fit | SQ (6) | Lag | 4 | 27% | *"It sometimes lags, displaying a 'system busy' prompt."* (P3) |
|  |  | Instability | 2 | 13% | *"Stability is low; response quality fluctuates."* (P12) |
|  | CON (3) | Exceeds Expectations | 1 | 7% | *"The diversity of perspectives in the answers exceeds my expectations."* (P6) |
|  |  | Over-Extension | 2 | 13% | *"Generated content sometimes expands excessively and deviates from the core topic."* (P10) |
|  | IQ (5) | Off-Topic Answers | 2 | 13% | *"Some answers are irrelevant to what I asked."* (P2) |
|  |  | False Information | 2 | 13% | *"It predicted a postgraduate entrance score of 405, which was highly misleading."* (P9) |
|  |  | Format Disorder | 1 | 7% | *"The formatting of generated articles is occasionally non-standard."* (P11) |
|  | TTF (4) | Fast Specialized Retrieval | 2 | 13% | *"In highly specialized fields, retrieval is faster and more targeted."* (P3) |
|  |  | Scenario-Based Query | 1 | 7% | *"By setting contextual questions, I obtain more targeted answers."* (P4) |
|  |  | Postgraduate School Search | 1 | 7% | *"During the postgraduate application period, it is more convenient than traditional databases."* (P9) |
|  | TEC (6) | Strong AI Comprehension | 3 | 20% | *"It understands the deeper meaning of questions and provides more insightful answers."* (P7) |
|  |  | Fluent Chinese | 2 | 13% | *"DeepSeek is more fluent in Chinese dialogue with stronger comprehension."* (P12) |
|  |  | Multimedia Output | 1 | 7% | *"I hope it will support PDF or video output in the future."* (P7) |
|  | TAC (3) | Review Keywords | 1 | 7% | *"When writing reviews, I need to quickly identify keywords."* (P9) |
|  |  | Precise Question Search | 1 | 7% | *"Final-exam practice requires precise question retrieval."* (P9) |
|  |  | Pharmaceutical Specialty | 1 | 7% | *"It offers new ideas in pharmaceutical research."* (P3) |
| Risk Perceptions | PC (4) | Concern about Fees | 3 | 20% | *"I worry that it will become paid in the future."* (P1) |
|  |  | Data Leakage | 1 | 7% | *"I fear my data might be misused; I need to verify it myself."* (P9) |
| User Outcomes | SAT (5) | Very Satisfied | 3 | 20% | *"I am very satisfied."* (P1) |
|  |  | High Rating | 2 | 13% | *"On a 10-point scale, I give it 9."* (P11) |
|  | CUI (14) | Continue if Free | 7 | 47% | *"I will keep using it as long as it remains free."* (P6) |
|  |  | Pay if Reasonable | 2 | 13% | *"If the pricing is reasonable and meets my needs, I would consider paying."* (P3) |
|  |  | Switch if better | 5 | 33% | *"If a better product becomes available, I would switch."* (P3) |
|  | CUB (2) | Daily Writing | 1 | 7% | *"I use it every day for paper writing."* (P10) |
|  |  | Final-Exam Search | 1 | 7% | *"I rely on it entirely for searching questions during finals."* (P9) |
